# An Overview of Ultraviolet-Protective Clothing

**DOI:** 10.7759/cureus.27333

**Published:** 2022-07-27

**Authors:** Jasmine T Lu, Erum Ilyas

**Affiliations:** 1 Dermatology, Drexel University College of Medicine, Philadelphia, USA; 2 Dermatology, Bryn Mawr Hospital, King of Prussia, USA

**Keywords:** uv protective clothing, uv protection, upf, sun protective clothing, sun protection

## Abstract

Protection from harmful ultraviolet radiation (UVR) can be achieved in a multitude of ways: sunscreen, chemical laundering additives, regular clothing, and photoprotective clothing. While sunscreen remains a popular sun-protective method, research has shown that its long-term use can lead to serious neurological, endocrine, and developmental consequences. Chemical laundering additives have been marketed as a means to absorb or reflect UVR, but data on its efficacy tests are not currently available, and skin contact with these chemicals may prove to be harmful. All clothing, regular or sun-protective, confers sun protection through dyes, weave patterns, and textile materials. However, photoprotective clothing is generally rated as more protective on the ultraviolent protection factor (UPF) scale and garment protection factor (GPF) scale. A combination of photoprotective clothing and sunscreen use over non-covered body areas is likely to be the optimal way for sun protection with minimal risk. However, further research on the topic is needed to gain deeper insights into it.

## Introduction and background

Skin cancer is the most common form of cancer in the US, with a higher annual incidence rate than all other cancer types combined [1]. As with all cancers, the risk of developing skin cancer increases with exposure to carcinogens. In the case of skin cancer, a major carcinogen is ultraviolet radiation (UVR) from the sun, with 86% of melanoma cases stemming from UVR exposure [2]. UVR can be further categorized into three different types based on their wavelengths: ultraviolet A (UVA), ultraviolet B (UVB), and ultraviolet C (UVC),. Though UVA rays can accelerate aging effects on the skin and UVB rays can cause sunburns, UVC is absorbed by the Earth’s atmosphere and therefore does not penetrate. Modern-day solutions to limit the damage caused by UVR include sunscreen and sun-protective clothing.

While sunscreen remains a popular protectant against the sun and has been shown to reduce melanoma incidence by 50% [3], some studies have suggested that prolonged, routine sunscreen absorption through the skin barrier is associated with neurotoxicity, as well as detrimental endocrine and developmental effects [4]. Experimental data based on studies in rats and zebrafish indicate that compounds present in sunscreen, such as benzophenones (BP), can cross the blood-brain barrier [4], potentially altering the tightly regulated composition of the cerebral spinal fluid surrounding the brain or the neuroendocrine system, which can have dangerous consequences. High concentrations of BP (up to 1000 μg/l) were found to impede hormones necessary for sexual reproduction in zebrafish. Rats that were given BP doses at a concentration and frequency (5 mg/kg/day for 30 days) similar to human exposure or at a lower dosage (0.1-10 μg/ml) did not demonstrate neurological or behavioral changes, but rat neural cell cultures exhibited less viability after exposure to BP in moderate concentrations (1-10 µg/ml). Furthermore, rats exposed to 4-methylbenzylidene camphor, another common sunscreen compound, at concentrations between 7-47 mg/kg were found to have impaired function of hormonal (androgen, estrogen, progesterone, thyroid hormone, etc.) activity, and altered expression of genes related to healthy development [4].

Another issue associated with sunscreen use is a lack of consistent application in users. A study done in Minnesota comparing the sunscreen habits of those diagnosed with melanoma and those who were melanoma-free found that, although the use of sunscreen with a sun protection factor (SPF) of 15+ was significantly higher in melanoma-free subjects, the mean lifetime usage of sunscreen was low in both groups [5]. The low sunscreen usage seems to suggest that some other factor is responsible for conferring extra protection to those who are melanoma-free. In the same study, the usage of “other” sun-protective methods, including wearing hats and long-sleeved shirts in addition to seeking shade, were reported to be practiced at a significantly higher rate by individuals who were melanoma-free than individuals diagnosed with melanoma. However, the study did not consider other risk factors for melanoma, such as a family history of melanoma or individual history of sunburns. Incidence of basal cell carcinomas (BCCs) and squamous cell carcinomas (SCCs) in relation to sun-protective methods was compared between groups of patients who used sunscreen daily and groups of patients who used sunscreen only occasionally; the incidence of BCCs and SCCs was found to be similar among both groups, although it was noted that the evidence was of “low quality” [6]. In recent years, clothing, especially those designed specifically to be “sun-protective”, has been more widely researched as a potential supplemental, yet necessary defense in addition to sunscreen.

Few studies have thoroughly investigated sun-protective clothing as a photoprotective method. Based on the scarce data that is available, the consensus is that sun-protective clothing is effective in reducing UVR transmittance to the skin, with its ability to block UVR graded on the ultraviolet protection factor (UPF) scale or, more recently and specifically, the garment protection factor (GPF) scale. A UPF rating of 15, 30, and 50+ correspond to clothing that blocks 93.3%, 96.7%, and 98% of UVR transmittance, respectively. To receive the Seal of Recommendation from The Skin Cancer Foundation, the fabric must achieve a UPF rating of 30 at a minimum, as tested and presented by the company seeking the Seal. However, the efficacy of sun-protective clothing has been challenged by multiple outside studies. One such study measured UVA and UVB transmittance levels of clothing marketed as UPF 50+ sun-protective vs. regular clothing composed of cotton and wool, concluding that there was markedly decreased UVR transmission through sun-protective and regular clothing alike [7]. Though the study argued that regular clothing, specifically wool items, was more effective in blocking UVR than the UPF 50+ sun-protective garments, the research failed to account for confounding factors such as the number of launderings for all clothing items and differences in the weaving patterns of the fabrics. As required by the authorities in the US, in order to receive the “UV Protective” label, the testing process must involve simulating two years of wear and tear on the garment before UV transmittance is measured [8]. Such conditions include 40 launderings to mimic the loss of clothing density after washing, as well as exposure to controlled sunlight and/or chlorinated pool water. Furthermore, chemical additives that may have been added to the articles of clothing used for testing need to be accounted for.

Despite the numerous confounding factors that may arise when attempting to determine the true value of sun-protective clothing, examining body surface area (BSA) as a parameter of UPF via the “hole effect” has gained increasing popularity. According to the “hole effect”, clothing is naturally porous, containing holes through which UVA and UVB are absorbed into the skin at 100% intensity [9]. A greater BSA exposed to the sun requires greater sunscreen application to achieve sun protection. The use of sun-protective clothing can likely significantly decrease BSA exposure and subsequently reduce the amount of sunscreen application required.

## Review

Currently available sun-protection methods related to clothing 

Regular Clothing

Clothing not specified as “sun-protective” is capable of blocking UVR with varying levels of success, depending upon specific dye and weave patterns. As per multiple research studies, garments that are dyed tend to provide more UV protection. Clothing with darker dyes, i.e. red, black, or navy blue, has repeatedly shown significantly lower UVR transmittance in comparison to clothing with lighter dyes, i.e., pastels or yellow, or clothing that is white [10,11]. However, in comparisons between light-dyed clothing and dark-dyed clothing, significantly higher UPF values were only found with a high intensity of colored dye; dark-dyed clothing that had a lower intensity of colored dye did not demonstrate significantly different UPF values than light-dyed clothing [12], suggesting that the quantity of dye was more indicative of UV-protective ability than the color of the dye. In line with this finding, The Skin Cancer Foundation has stated that clothing with either dark or bright dyes absorbs comparatively more UVR than clothing with lighter dyes or no dye at all [13].

Different fabric materials exhibit varying abilities in minimizing UVR transmittance according to the tightness of their weave. In one study, linen, one of the most porous fabrics, demonstrated the lowest UPF values, followed closely by knitted fabrics. On the other hand, polyester fabrics, including polyester and cotton blends, and fabrics used in polo shirts all achieved excellent UPF ratings, well above a UPF of 50 [12]. The success of the polyester garments and polo shirts in reducing UVR transmittance has been attributed to low fabric porosity, further implying that clothes with higher coverage of BSA can act as a greater defense against sun exposure and therefore reduce the need for sunscreen application.

*Chemical Laundering Additives* 

There are two common photoprotective laundry additives commercially available for conferring additional sun protection to clothing when added during the laundering process: Rit Sun Guard and optical brightening agent (OBA). Rit Sun Guard, recommended by The Skin Cancer Foundation, functions by sticking to clothing fibers and absorbing UVR via its active ingredient, TINOSORB FD. A single laundering session using Rit Sun Guard supposedly grants clothing a UPF of 30 for up to 20 washes afterward [14]. However, data on how the UPF rating for Rit Sun Guard was determined or if its effects truly last for up to 20 post-additive launderings is currently lacking. OBA, another common UV absorber, works by essentially bleaching garments such that UV rays are absorbed and then re-emitted [9]. As is the case with Rit Sun Guard, little data is available on the photoprotective efficacy of OBA.

*UV Textiles* 

UV textiles have recently gained traction as another effective method for limiting UVR exposure. To achieve high UPF values, photoprotective clothing is specifically designed with effective textile composition and dying patterns in mind rather than using chemical additives. UV textiles tend to be composed entirely of synthetic fibers such as polyester and nanofibers, which are woven tightly together and permit far less direct skin exposure to UVR as conferred by the “hole effect” [12]. Furthermore, UV-protective clothing typically utilizes clothing dyes, especially dyes with brighter colors, as clothing with brighter colors have been reported to reflect UVR by The Skin Cancer Foundation [13]. There exist multiple brands that currently market UV-protective clothing based on such principles, including AmberNoon, Coolibar, and Solbari. However, given that photoprotective clothing has only just emerged, more research is needed to determine its true efficacy. 

Challenges

Carcinogenic Chemical Additives

Though chemical additives for clothing are intended to serve protective purposes, the chemicals themselves can pose a carcinogenic risk as evidenced by higher cancer rates observed among firefighters. Polybrominated diphenyl ethers (PBDEs), which are found to be carcinogenic and neurotoxic in studies using animal subjects [15], are part of the flame-retardant chemical additive used to coat firefighter personal protective equipment (PPE), such as jackets and trousers [16]. Samples collected from firefighter PPE, including unused PPE articles, have demonstrated that firefighters are exposed to higher levels of PBDE when compared to the general public [17]. Furthermore, PBDEs persist even after multiple launderings [18], meaning that dermal exposure to PBDE occurs over a long period of time for firemen. The carcinogenic chemical hazards faced by unaware firemen greatly highlight the need for sun-protective chemical additive companies to disclose how their products achieve sun protection and/or the chemical make-up of such products to ascertain a lack of danger.

UPF vs. GPF Scaling

As research has come to focus more on investigating sun-protective clothing, the GPF scale is increasingly standardized for the rating of UV-protective clothing in place of the UPF scale. Unlike the UPF scale, the GPF scale takes into account both UPF potential and BSA coverage, acknowledging the role that BSA exposure plays in conferring UVR resistance. With a range of 0-6+, the GPF scale specifies a “minimum”, “good”, and “excellent” rating for clothing scoring 0-3, 3-6, and 6+, respectively. By experimentally fitting sun-protective clothing onto a mannequin and determining UVR transmittance with respect to both UPF and BSA, one study found that photoprotective clothing could be awarded a higher UPF rating if BSA coverage was adequate [19]. Such findings strongly suggest that BSA is an important factor when considering the ability of sun-protective clothing to decrease skin exposure to UVR and, therefore, skin cancer risk. Thus, while less widely known and used, the GPF scale is more comprehensive in its rating approach than the currently popular UPF scale.

## Conclusions

Adequate sun protection and limiting UVR transmittance are associated with several known health benefits, including the prevention of life-threatening skin cancer. While a popular sun-protective method involves applying sunscreen, large amounts of sunscreen absorbed dermally have been found to cause detrimental neurotoxic, endocrine, and developmental defects. In addition, the practice of using sunscreen is generally low among the public. Photoprotective methods such as chemical laundering additives and UV textiles have emerged, with effectiveness in blocking UV rays as rated on the UPF scale. Though certain chemical additives, i.e., Rit Sun Guard, have been endorsed by The Skin Cancer Foundation as effective in absorbing UVR, the chemical make-up of such additives remains unknown and further research is needed to confirm that these products do not pose unforeseen health risks. UV textiles generally provide photoprotection by using synthetic, tightly woven polyester and/or nanofibers, as well as dying clothing in darker or brighter colors. However, due to the fact that UV-protective clothing is of recent origin, few studies have been conducted to investigate its efficacy. The ongoing transition from using the UPF scale to using the GPF scale, which takes into consideration BSA during the determination of photoprotective quantity, suggests that sun protection may be best achieved by using a combination of UV textiles and sunscreen, with the former covering a significant portion of the body surface and the latter protecting the remaining body regions exposed to UVR.
